# Biomarkers of Endothelial Damage in Distinct Phases of Multisystem Inflammatory Syndrome in Children

**DOI:** 10.3390/metabo12080680

**Published:** 2022-07-24

**Authors:** Monica Gelzo, Antonietta Giannattasio, Marco Maglione, Stefania Muzzica, Carolina D’Anna, Filippo Scialò, Thaililja Gagliardo, Michela Grieco, Vincenzo Tipo, Giuseppe Castaldo

**Affiliations:** 1 CEINGE-Biotecnologie Avanzate, Scarl, 80145 Naples, Italy; monica.gelzo@unina.it (M.G.); filippo.scialo@unicampania.it (F.S.); 2 Dipartimento di Medicina Molecolare e Biotecnologie Mediche, Università di Napoli Federico II, 80131 Naples, Italy; 3 Pediatric Emergency and Short Stay Unit, Santobono-Pausilipon Children’s Hospital, 80129 Naples, Italy; antonella.giannattasio@virgilio.it (A.G.); marcomaglione84@gmail.com (M.M.); stefaniamuzzica@hotmail.it (S.M.); dannacarol@alice.it (C.D.); thaililja@virgilio.it (T.G.); michelagrieco@tiscali.it (M.G.); v.tipo@santobonopausilipon.it (V.T.); 4 Dipartimento di Medicina Traslazionale, Università della Campania L. Vanvitelli, 80131 Naples, Italy

**Keywords:** endothelial hyperinflammation, MCP-1, MIS-C, pANCA, vasculitis, VEGF-A

## Abstract

Endothelial hyperinflammation and vasculitis are known hallmarks of acute COVID-19 and multisystem inflammatory syndrome in children (MIS-C). They are due to the direct effect of the virus on endothelial cells enhanced by pro-inflammatory modulators and may cause venous/arterial thrombosis. Therefore, it is essential to identify patients with endothelial damage early in order to establish specific therapies. We studied the monocyte chemoattractant protein 1 (MCP-1), the perinuclear anti-neutrophil cytoplasmic antibodies (pANCA), and the vascular endothelial growth factor A (VEGF-A) in serum from 45 MIS-C patients at hospital admission and 24 healthy controls (HC). For 13/45 MIS-C patients, we measured the three serum biomarkers also after one week from hospitalization. At admission, MIS-C patients had significantly higher levels of MCP-1 and VEGF-A than the HC, but no significant differences were observed for pANCA. While after one week, MCP-1 was significantly lower, pANCA was higher and VEGF-A levels were not significantly different from the admission values. These findings suggest an involvement of epithelium in MIS-C with an acute phase, showing high MCP-1 and VEGF-A, followed by an increase in pANCA that suggests a vasculitis development. The serum biomarker levels may help to drive personalized therapies in these phases with anticoagulant prophylaxis, immunomodulators, and/or anti-angiogenic drugs.

## 1. Introduction

The endothelial hyperinflammatory involvement is a known hallmark of acute COVID-19 infection [1] and is due to the direct effect of the virus on endothelial cells [2] enhanced by pro-inflammatory modulators [3]. It may cause venous and arterial thrombotic complications [3]. Thus, it is critical to identify early the endothelial damage in COVID-19 patients in order to establish the need of anticoagulant prophylaxis. Serum endothelial biomarkers such as selectins [4] and MCP-1 [5] may help to reveal patients with a higher risk. In some patients with acute COVID-19 infection, the endothelial damage may appear as a systemic vasculitis associated with the production of pANCA [6], and such a condition was recently found also in post-COVID children [7].

Multisystem inflammatory syndrome in children (MIS-C) is a rare, severe complication of COVID-19 [8,9] that appears 2 to 6 weeks after SARS-CoV-2 acute infection. Although its pathogenesis is still undetermined, it may represent a delayed, hyperimmune response to SARS-CoV-2 [10]. Among non-specific symptoms such as fever, and gastrointestinal and neurologic alterations, severe complications including cardiovascular shock and multi-organ failure appear in a percentage of MIS-C patients [11,12,13]. Vasculitis and microthrombosis, particularly at the pulmonary level, were observed by necroscopy in MIS-C patients [14]. On the other hand, MIS-C shares some clinical features with other pediatric inflammatory multisystemic syndromes, for example, Kawasaki Disease (KD), toxic shock syndrome (TSS), macrophage activation syndrome (MAS), hemophagocytic lymphohistiocitosis (HLH), and others [15,16,17] typically associated with endothelial damage and systemic vasculitis. While in Asian countries no cases of MIS-C were reported [18], in Western countries MIS-C represents a critical health condition associated with SARS-CoV-2 infection.

A better knowledge of the vascular damage in MIS-C patients would help to cast light on the pathogenesis of this disease, and it would help to reveal specific diagnostic biomarkers and/or develop targeted therapies. Toward this aim, we studied in serum from MIS-C patients, different phases of the disease and three biomarkers related to endothelium damage, i.e., (i) monocyte chemoattractant protein 1 (MCP-1), a chemotactic molecule that is released by various cells among which endothelium after vascular endothelium injury. It attracts and activates cells among which monocytes, promoting inflammation, and thromboembolic events, and was recently found increased in serum from severe COVID-19 patients [19]; (ii) perinuclear anti-neutrophil cytoplasmic antibodies (pANCA), specifically addressed against neutrophil myeloperoxidase, previously found enhanced in the serum of COVID-19 acute patients [6] and in pediatric post-COVID vasculitis [7]; and (iii) vascular endothelial growth factor A (VEGF-A), a biomarker of pathological neo-angiogenesis [20].

## 2. Results

**Figure 1:**
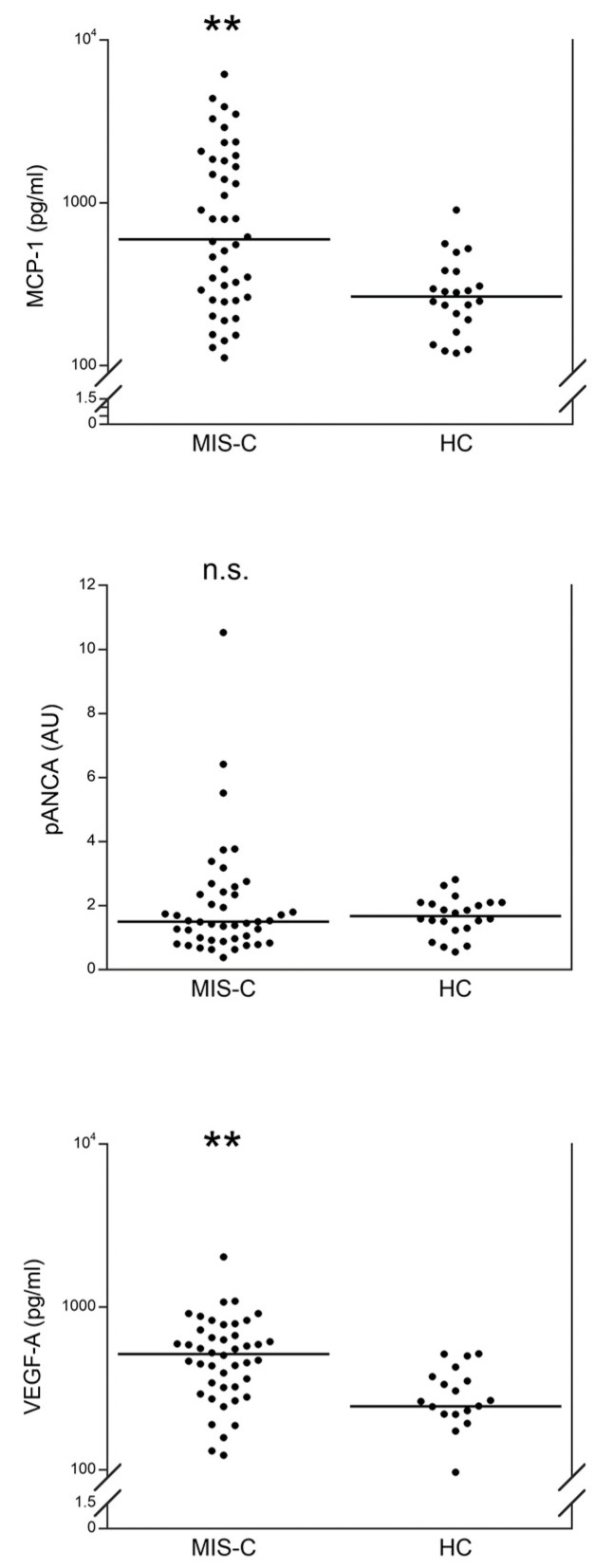
Comparison of serum values of MCP-1, pANCA, and VEGF-A in patients with MIS-C and in healthy controls (HC) at hospital admission. The line represents the median value. ** *p* < 0.001; n.s.: not significant.

Figure 1 shows the serum levels of MCP-1, pANCA, and VEGF-A in 45 patients with MIS-C at diagnosis and in 24 HC. Levels of MCP-1 were significantly higher in MIS-C patients than HC (*p* < 0.001), with 12 out of 45 (26.7%) MIS-C patients having >1000 pg/mL (the highest MCP-1 level in HC). No significant differences were observed between MIS-C patients and HC for serum pANCA. While, VEGF-A serum levels were significantly higher in MIS-C patients than HC (*p* < 0.001), with 6 out of 45 (13.3%) MIS-C patients having >800 pg/mL (the highest VEGF-A level in the HC).

Table 1 reports the results of the Spearman correlation analysis in MIS-C patients at admission. Serum MCP-1 levels were positively associated with serum levels of IFNγ and IL-6. While an inverse correlation has been found for MCP-1 versus pANCA and the number of monocytes.

**Figure 2:**
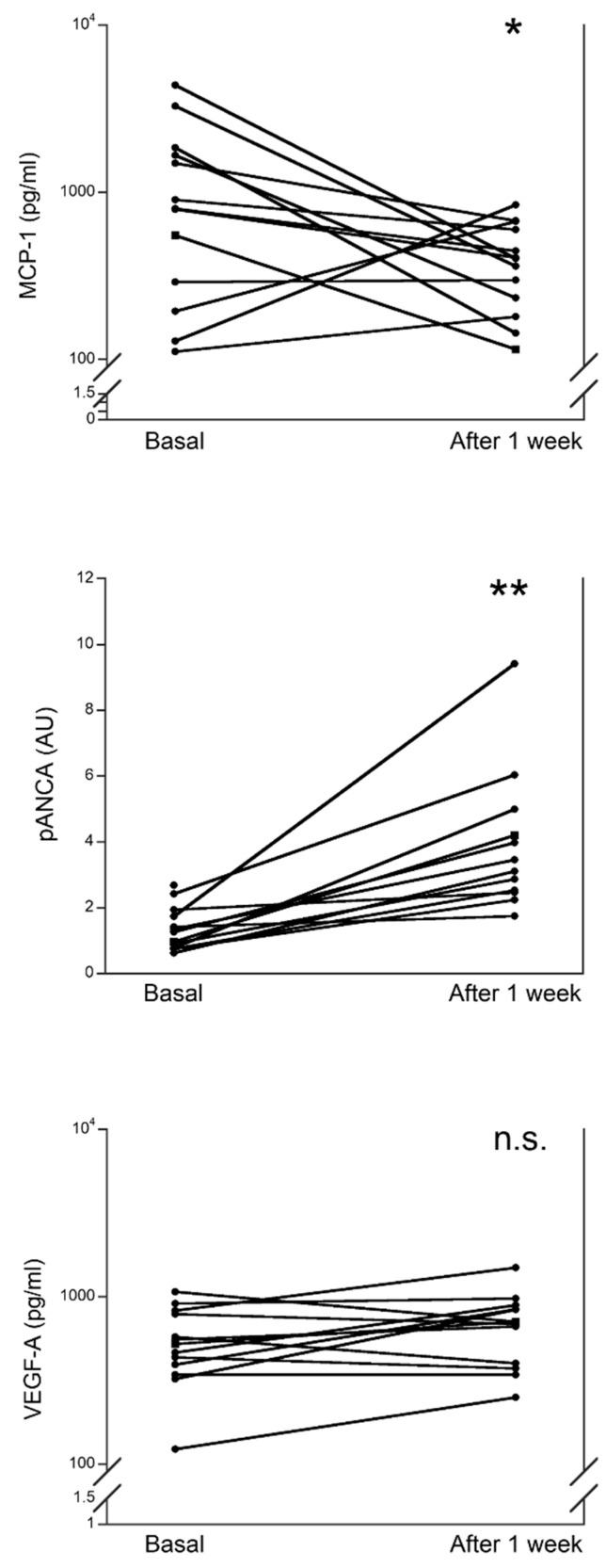
Comparison of serum levels of MCP-1, pANCA, and VEGF-A in patients with MIS-C at hospital admission and after one week of hospitalization. * *p* < 0.01; ** *p* < 0.001; n.s.: not significant.

For 13/45 MIS-C patients, we measured serum MCP-1, pANCA, and VEGF-A at diagnosis and after one week from hospitalization (Figure 2). In these patients, the baseline levels of the three serum biomarkers were comparable to those in the MIS-C patients that have been discharged from the hospital before one week. After one week of hospitalization, the levels of serum MCP-1 were significantly lower (*p* < 0.01), although in the three patients with the lowest levels of MCP-1 we observed an increase. On the other hand, the levels of pANCA were significantly higher after one week of hospitalization (*p* < 0.001). For serum VEGF-A, no significant differences were observed between the levels at admission and after one week and discordant trends were observed that cannot be associated with the baseline levels.

## 3. Discussion

We report that patients with MIS-C at hospital admission had a significant increase in serum MCP-1 and VEGF-A but not for pANCA, as compared to the HC. While at one week from the admission, serum levels of MCP-1 were significantly reduced as compared to the values at admission and were not significantly different from the HC; the levels of pANCA were significantly higher as compared to the values at admission and to the HC; and the levels of VEGF-A were not significantly different as compared to the evaluation at admission. 

Our results are comparable with previous studies performed on patients with acute COVID-19 infection and with MIS-C. In particular, an increase of three folds of serum MCP-1 was found in adult critical patients with acute COVID-19, reflecting the hyperinflammatory endothelial dysfunction [1]. An increase in serum MCP-1 was observed in acute COVID-19 and even more in MIS-C children [21], and another study reported the increase in serum MCP-1 in MIS-C patients with the reversion to normal levels after methylprednisone treatment by the fifth day of the disease [22]. However, the reversion of serum values of MCP-1 cannot exclude potential damages induced by this molecule. In fact, the overexpression of MCP-1 during MIS-C attracts various immune cells among which are monocytes, which are the main drivers of inflammation in patients with MIS-C with microvascular alterations, particularly at the pulmonary level [23]. In agreement with these data, we found a significant positive correlation between serum MCP-1 levels and the serum levels of IFNγ and IL-6, biomarkers of type II IFN activation and cytokine responses. The targeting of MCP-1 is a potential therapeutic approach in a series of human disorders that includes multiple sclerosis, obesity-associated inflammation, and cancer metastasis [24]. Thus, considering that one third of patients with MIS-C had serum values of MCP-1 > 1000 pg/mL, we hypothesize, for these cases, a targeting treatment of MCP-1. 

Among the 45 MIS-C patients, 13 patients remained in the hospital after one week. They can be considered representative of the MIS-C patients, as no differences have been observed between the baseline data of these patients and those that have been discharged from the hospital before one week. In these patients, after one week from hospitalization, we found a significant reduction of MCP-1, although the patients with the lowest levels of MCP-1 at diagnosis showed an increase in this biomarker after one week. This could be due to an earlier diagnosis in these patients and therefore to a later acute phase. In addition, after one week, we found an increase in serum pANCA, that was inversely correlated with serum MCP-1 at hospital admission. These antibodies are addressed against neutrophil myeloperoxidase and their production may be triggered by the enhanced apoptosis of neutrophils [25], also induced by neutrophil extracellular traps [26]. Among our study population, an 8 years-old female patient showed a five-fold increase in pANCA after 1 week and she had a severe myocarditis requiring inotropes and pediatric intensive unit care admission. Serum pANCA is a biomarker of vasculitis that may appear with a wide range of clinical phenotypes including severe glomerulonephritis, recently described in two adults [27] and in a pediatric patient [7] after COVID-19. Serum pANCA may also be associated with less severe vasculitis [25] with milder symptoms [28]. In fact, we recently described a transient increase in serum pANCA in patients with SARS-CoV-2 infection, suggesting subclinical vasculitis or an aspecific epiphenomenon of the infection with no clinical relevance [6]. However, among the form associated to serum pANCA is included small vessel necrotizing vasculitis involving the lungs [29]. Most studies report systemic vasculitis in MIS-C patients [2,30], similarly to KD and HLH [31] that share several clinical similarities with MIS-C. However, no studies tested for pANCA in patients with MIS-C so far. We suggest performing such a test in patients with MIS-C and to monitor carefully patients with higher values of serum pANCA. 

Finally, we found an increase in serum VEGF-A in patients with MIS-C at hospital admission, and this increase was maintained at one week from hospitalization. Serum VEGF-A is a marker of an abnormal process of re-vascularization that occurs in patients with neoplasia, chronic inflammation, or other severe disorders [16]. An increase in such a biomarker in the serum from patients with MIS-C, already found in previous studies [20,32], strongly suggests further investigating the angiogenic processes associated with MIS-C, including the use of anti-angiogenic therapies in patients with the highest levels of serum VEGF-A.

A study limitation is represented by the lack of biomarkers analyses after one week for all enrolled MIS-C patients due to the transfer of patients to other hospitals once the more severe phase was resolved and the therapy defined. Another limitation is the lack of confirmatory analyses, such as the investigation of apoptosis biomarkers to confirm if the increase in pANCA antibodies could be due to neutrophil apoptosis. Therefore, these preliminary results need to be confirmed by further investigations.

## 4. Materials and Methods

### 4.1. Patients

We enrolled 44 children at hospital admission diagnosed as MIS-C according to the definition of the Centre for Disease Control and Prevention [32,33]. The study was approved by the Ethical Committee of the University Federico II of Naples. All procedures conformed to the Declaration of Helsinki. Informed consent was obtained from the parent/guardian. The only exclusion criterion was the impossibility to obtain consent (*n* = 0). MIS-C patients had a median age of 7 years (range: 1–14 years) and 17/44 (39%) patients were females. All patients started specific treatment (intravenous immunoglobulins together with high or low dose of steroids according to severity) within 48 h since hospital admission. After incomplete response to initial therapy with a lack of clinical and laboratory response and a worsening of inflammation parameters, anakinra was administered in 5 cases with rapid clinical and laboratory improvements. The healthy control (HC) group included 24 age- and sex-matched children (median age: 9 years; age range: 5 months–16 years; females, %: 42%).

### 4.2. Blood Cell and Serum Biomarker Analyses

Blood samples were collected in tubes containing EDTA and then immediately analyzed by flow cytometry, and in tubes without anticoagulant and then centrifuged for serum biomarker analysis. The numbers of neutrophil, monocytes, and T lymphocytes were measured by multicolour flow cytometry (Facs Canto II; Becton Dickinson Italia, Milan, Italy) as previously described [34]. The levels of serum MCP-1, pANCA, VEGF-A, IFNγ, and IL-6 were analyzed by automated microfluidic immunoassay cartridges on ProteinSimple Ella (Bio-Techne, Minneapolis, MN, USA), in accordance with the manufacturer’s instructions.

### 4.3. Statistical Analyses

The Shapiro–Wilk test was applied to evaluate the normality of distributions. Comparisons between the two groups were evaluated by the Mann–Whitney U test. Correlations between variables were evaluated using Spearman’s correlation analysis. For the statistical analysis of values below the limits of sensitivity, concentrations were estimated using the formula of limit of sensitivity/√2 [35]. Paired comparisons between basal and after 1-week values were performed by Wilcoxon signed rank test. Linear regression analysis was used to assess the effect of age and gender (independent variables) on MCP-1, pANCA, and VEGF-A (dependent variables) by stepwise method. Statistical analyses were performed by SPSS (version 27, IBM SPSS Statistics, NY, USA). Graphics were done using KaleidaGraph software (version 4.5.4, Synergy, Reading, PA, USA). *p* values < 0.05 were considered significant.

## 5. Conclusions

This study suggests an involvement of epithelium in MIS-C with an acute phase that shows high MCP-1 and VEGF-A. In fact, the levels of serum MCP-1 significantly correlated with serum levels of IFNγ and IL-6. This acute phase is followed by an increase in pANCA that suggests vasculitis development. The levels of serum biomarkers may help to drive personalized therapies in these phases with anticoagulant prophylaxis, immunomodulators, and/or anti-angiogenic drugs.

## Figures and Tables

**Table 1:** Spearman correlation analysis in MIS-C patients.

Variables	MCP-1 (pg/mL)		pANCA (AU)		VEGF-A (pg/mL)	
	r_s_	*p* Value	r_s_	*p* Value	r_s_	*p* Value
MCP-1 (pg/mL)	-	-	−0.421	**0.005**	−0.073	0.642
pANCA (AU)	−0.421	**0.005**	-	-	0.198	0.202
VEGF-A (pg/mL)	−0.073	0.642	0.198	0.202	-	-
IFNγ (pg/mL)	0.665	**<0.0001**	−0.325	0.065	−0.102	0.572
IL-6 (pg/mL)	0.684	**<0.0001**	−0.317	0.072	−0.008	0.967
Neutrophils (N/mmc)	0.089	0.583	0.157	0.333	0.288	0.072
Monocytes (N/mmc)	−0.371	**0.018**	0.277	0.083	−0.012	0.941
T lymphocytes (N/mmc)	−0.201	0.214	0.037	0.820	−0.064	0.693

Significant values are reported in bold. R_s_: Rho di Spearman.

## Data Availability

The data presented in this study are available in article.
